# Online peer support for people with Amyotrophic Lateral Sclerosis (ALS): a narrative synthesis systematic review

**DOI:** 10.3389/fdgth.2024.1138530

**Published:** 2024-01-31

**Authors:** Esther Vera Gerritzen, Abigail Rebecca Lee, Orii McDermott, Neil Coulson, Martin Orrell

**Affiliations:** ^1^ Institute of Mental Health, Mental Health and Clinical Neuroscience, School of Medicine, University of Nottingham, Nottingham, United Kingdom; ^2^ Population and Lifespan Health, School of Medicine, University of Nottingham, Nottingham, United Kingdom

**Keywords:** Amyotrophic Lateral Sclerosis, motor neuron disease, online health community, online peer support, narrative synthesis

## Abstract

**Background:**

Amyotrophic Lateral Sclerosis (ALS) significantly impacts the lives of people with the diagnosis and their families. A supportive social environment is important for people with ALS to adopt effective coping strategies and health behaviours, and reduce depressive symptoms. Peer support can provide a supportive social environment and can happen in-person and online. Advantages of online peer support are that people can engage from their own home, at their own time and pace, and that it offers a variety of different platforms and modes of communication.

**Objectives:**

To (1) explore the benefits and challenges of online peer support for people with ALS, and (2) identify successful elements of online peer support for people with ALS.

**Methods:**

The method selected for this systematic review was a narrative synthesis. Six databases were systematically searched in April 2020 for articles published between 1989 and 2020. The search was updated in June 2022. The quality of the included studies was assessed with the Critical Appraisal Skills Programme qualitative research checklist.

**Results:**

10,987 unique articles were identified through the systematic database search. Of those, 9 were included in this review. One of the main benefits of online peer support was that people could communicate using text rather than needing verbal communication, which can be challenging for some with ALS. Successful elements included using profile pages and graphics to identify others with similar or relevant experiences. Challenges included ALS symptoms which could make it difficult to use technological devices.

**Conclusions:**

Peer support can provide a non-judgmental and supportive environment for people with ALS, in which they can exchange experiences and emotional support, which can help people in developing adaptive coping strategies. However, ALS symptoms may make it more difficult for people to use technological devices and engage in online peer support. More research is needed to identify what kind of specific barriers people with ALS experience, and how these could be overcome.

## 1 Introduction

### 1.1 Background

Amyotrophic Lateral Sclerosis (ALS) is a motor neuron disease that affects the lower and upper motor neurons. ALS progresses rapidly, with most people living 3–5 years after they get diagnosed (1). Typically, people are around 60 years old when they get diagnosed (2). ALS is a rare condition and there are few recent studies on the epidemiology of ALS. A 2019 Global Burden of Motor Neuron Disease study estimated that in 2019 around 268,000 people were living with motor neuron disease globally. When looking at regions in the world, this study shows that the highest prevalence of Motor Neuron Disease is in Western Europe, with more than 56,000 people living with the condition in 2019. This is followed by Tropical Latin America (including Brazil and Paraguay) with over 44,000 people, and North America and East Asia with both over 43,000 people. The same study found that in 2019, Motor Neuron Disease caused more than 1 million disability adjusted life years worldwide (3). As the condition progresses people can experience difficulties with speaking, eating, moving and breathing (1). Additionally, people with ALS can experience forms of cognitive impairment, including difficulties in recognising emotions in others, interpreting social situations (4), and apathy. Due to the nature of symptoms and the rapid progression of the condition, people with ALS need ongoing care and support (5).

ALS significantly impacts the lives of people living with the diagnosis and their families (5–7). Besides the physical symptoms, ALS also has an emotional impact. People with ALS can experience an increasing loss of control and dependency on others and often fear being a burden (5–8), as they have to rely on others for medication, personal care, and attending healthcare appointments (7). Matuz, Birbaumer (6) emphasize the importance of social support and adaptive coping strategies in adjusting to living with ALS. They found that higher levels of perceived social support and coping skills can reduce depressive symptoms. A supportive social environment without judgement is important to help people with ALS use effective coping strategies, reduce the impact of stress, and adopt positive health behaviours (6). Moreover, Matuz, Birbaumer (6) found that quality of life in people living with ALS is not mainly determined by the time since diagnosis or severity of symptoms, but more so by psychosocial factors. This is in line with the Social Health Framework, which states that health is about finding a balance between the limitations that someone experiences because of their health condition, and the abilities that they still have (9). For example, Dröes, Chattat (10) found that by focusing on one's abilities and positive coping strategies, people with dementia can still live meaningful and satisfying lives and perceive a good quality of life. For this, people need a strong social network that supports them to adapt and self-manage, and enables them to remain independent and autonomous for as long as possible (10). The Social Health Framework consists of three dimensions: the ability to (1) fulfill one's potential as well as obligations, (2) manage one's own life with some independence, and (3) participate in work and social activities (9).

One way to improve the Social Health of people with ALS is through peer support. Peer support is well-known for offering a non-judgmental environment where people who have similar life experiences or a similar health condition can exchange experiences and support (11). One of the characteristics of peer support is that there is reciprocity of support. Being in an environment where one can both receive and provide support to others can increase feelings of empowerment (11–13). Another characteristic of peer support is sharing experiential knowledge, which is the knowledge that people have because of their own experiences of living with a health condition. This can support people in developing new and positive coping skills (13). These characteristics are unique to peer support, and it shows that peer support can go beyond support that is available from healthcare professionals and friends or family who do not have an ALS diagnosis (14).

Peer support can happen in-person and in online settings. Online peer support includes a wide variety of different platforms, which have different modes of communication. For example, asynchronous (not in real time) communication on discussion forums or social media platforms can include text-based communication, but also communication through emoticons, images, and videos. Through videoconferencing platforms people can communicate verbally in real time. One of the main advantages of online peer support is that it is not limited by geographical barriers (15), making it potentially suitable for those who have rare conditions, do not have access to in-person support in their local area, or cannot travel. Additionally, online platforms can allow for anonymity, making it potentially suitable for people with stigmatised conditions (16) or to discuss taboo topics (17).

Research suggests that online peer support can be beneficial for people with chronic (18) or neurodegenerative (19, 20) conditions. Research on online peer support for people with ALS is growing and suggests it could be beneficial (see Stewart Loane and D’Alessandro (21) and Caron and Light (22) for examples). Weeks, Gould (7) found that people with ALS are interested in online peer support due to its convenience. For example, because people can engage with it when it suits them, and they do not have to rely on anyone to travel to an in-person meeting (7). However, knowledge on how online peer support may impact health outcomes and self-management for people with ALS is limited.

### 1.2 Objectives

This review aims to (1) explore the benefits and challenges of online peer support for people with ALS, and (2) identify successful elements of online peer support for people with ALS. Successful elements of online peer support are considered to be those aspects that can result in positive outcomes for the user. Challenges are those aspects that can make it more difficult for a person with ALS to use online peer support. This can relate to the technology as well as ALS symptoms. Understanding successful elements and challenges can be helpful in improving existing and developing new online peer support opportunities for people with ALS, as well as other chronic, neurodegenerative conditions.

## 2 Methods

This review followed the narrative synthesis procedures of Popay, Roberts (23) including four elements: (1) theory development (covered in background section), (2) development of a preliminary synthesis, (3) exploration of relationships in the data, and (4) assessment of the robustness of the synthesis. With the narrative synthesis method the findings are words- and text-based, making it a helpful method to identify best practices (23). This review is presented following the PRISMA 2020 guidelines (24).

### 2.1 Search strategy

A systematic database search was conducted in April 2020 and updated in June 2022. The search was part of a wider appraisal of the literature on different chronic neurodegenerative conditions (19, 20). Six databases were searched: CINAHL, Cochrane Library, Embase Medline, PsycINFO, Scopus, and Web of Science. The search terms and search filters that were used are presented in the Supplementary Material. One search filter regarding year of publication was applied (1989–2020) as the World Wide Web was introduced in 1989 (25). The filter on the year of publication was adjusted to 2020–2022 when the search was rerun. To reduce the risk of selection and publication bias, EVG conducted a search on Google Scholar and manually searched the reference lists of the included papers (26, 27). This did not result in new papers being added.

### 2.2 Inclusion and exclusion criteria

Inclusion criteria:

• The study population included people living with ALS or a blend of people with ALS and caregivers;
• The intervention included online peer support. Online peer support was regarded as communication via the Internet between peers in an online environment that is designed to facilitate social contact using either an asynchronous or synchronous text or text/video-based platform (e.g. social media platforms, forums, chat rooms or videoconferencing platforms);
• Publication between 1989 and 2020;
• Publication in peer reviewed journals.

Exclusion criteria:

• The study focused solely on caregiver perspectives;
• The intervention included online peer support that was part of a programme that also included in-person or telephone-based peer support;
• The study did not report on peer-to-peer interactions.
• They reported findings of literature reviews, opinion pieces, protocols, editorials, conference abstracts, or theses/dissertations;
• Papers were written in a language other than English if a translation was not available.

### 2.3 Study selection

The search results were imported into Endnote and duplicates were removed. EVG reviewed each title and abstract against the eligibility criteria which was followed by a full-text analysis of the potentially relevant studies. ARL provided a second independent review on studies labelled “unsure” in both stages. The main reason for labelling a study as unsure was that it met the eligibility criteria, but the outcomes did not mainly focus on peer-to-peer interactions (but rather on, for example, quality of life). Following a discussion with a senior member of the team (OM) the exclusion criteria were amended to exclude studies that did not report on peer-to-peer interactions. The papers that were included up until that point were reassessed.

### 2.4 Data extraction

EVG extracted the data using standardized data extraction forms including study information, study characteristics, population characteristics, characteristics of the online platform, outcomes, and implications for future research. ARL provided a second independent review of the completed data extraction forms.

### 2.5 Quality assessment

The Critical Appraisal Skills Programme (CASP) checklist for qualitative studies (28) was selected to assess the quality of the included studies. The CASP checklist was recommended in the Centre for Reviews and Dissemination guidance for undertaking reviews in healthcare (29). EVG completed the initial quality assessment and ARL provided a second independent review. The CASP checklist consists of 10 questions related to “rigour, credibility and relevance” (29). Studies were graded “high” if they met or partially met 8–10 items, “medium” if they met or partially met 5–7 items, and “low” if they met or partially met 0–4 items (30).

## 3 Results

**Figure 1:**
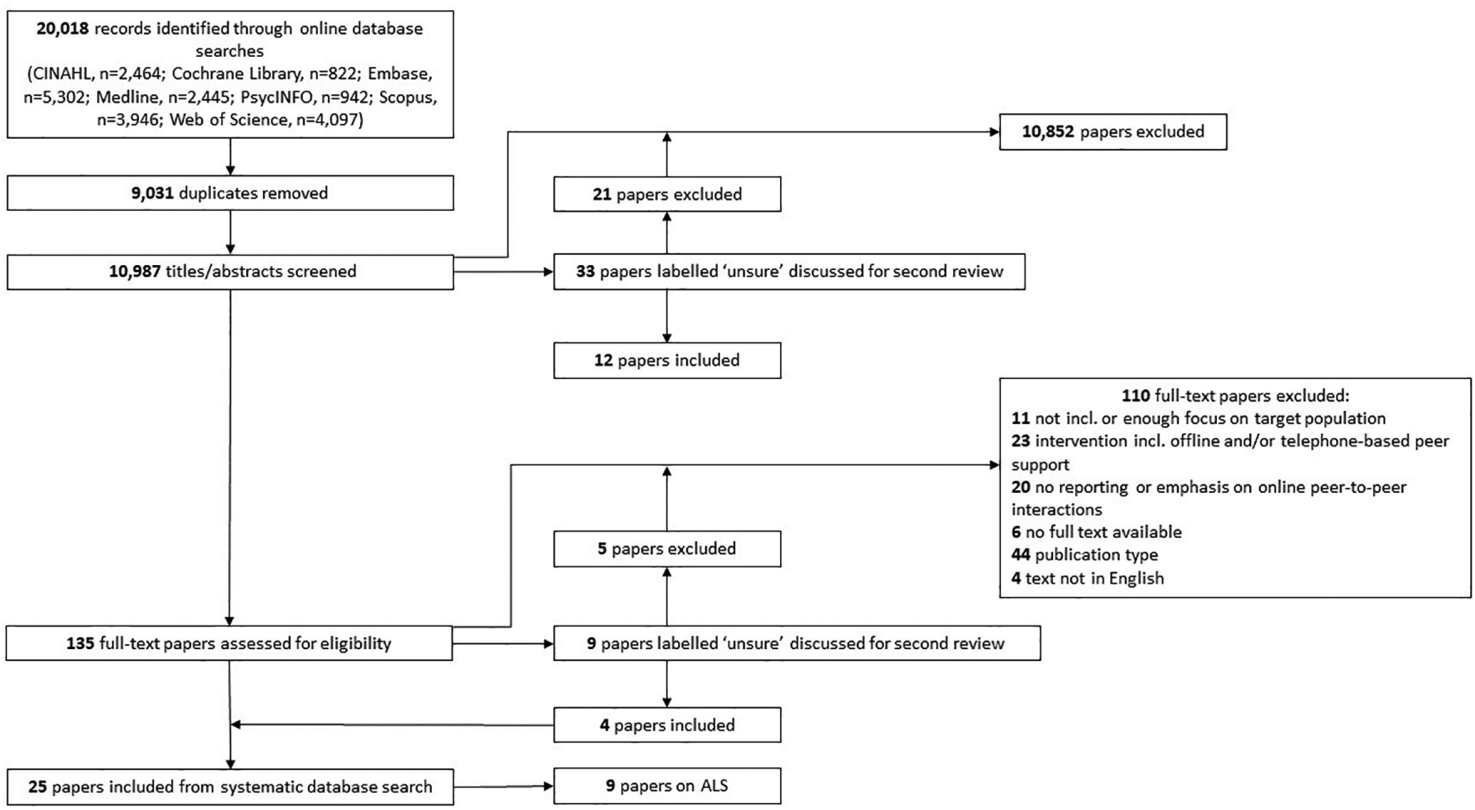
PRISMA (Preferred Reporting Items for Systematic Reviews and Meta-Analyses) diagram of the search and review process.

The results section covers element 2 of a narrative synthesis: developing a preliminary synthesis. The online database search returned 10,987 unique papers, of which nine were included (Figure 1). The updated search in June 2022 did not result in new studies being included.

### 3.1 Study characteristics

All studies included in this review used a qualitative design. The most frequently used method was content analysis (21, 31–36). Other methods include an asynchronous online focus group (22), interviews (33), and a case study (34). One study included people with ALS and carers (21) whereas the others only included people living with ALS (Table 1).

**Table 1:** Study characteristics.

Study (author, year)	Aim(s)	Design (methods)	Intervention	Setting (country)	Study population	Eligibility criteria	Sample size/participants	Quality assessment score
Stewart Loane and D'Alessandro (21)	Communication in an online ALS community (carers and patients)	Qualitative (content analysis)	Discussion forum	Unknown	People living with ALS and carers	Members from the ALS community selected for this study and their posts	133 members	9 (high)
							61 threads	
							499 posts	
Stewart Loane et al. (35)	Social support and consumer value in online health communities	Qualitative (content analysis)	Discussion forum	Unknown	People with ALS	2 online health communities, one for PD and one for ALS	PD community: 35 members, 30 threads, 137 posts	10 (high)
							ALS community: 133 members, 61 threads, 499 posts.	
Versteeg and te Molder (36)	Balance between expert advice and patient experiences	Qualitative (content analysis)	Discussion forum	Netherlands	People with ALS	Dutch online forums	1 patient support forum for ALS	5 (medium)
							20 threads	
Hargreaves et al. (33)	Empathy in discussion forums	Qualitative (interviews and content analysis)	Discussion forum	UK	People living with MND (ALS)	Open access forums for MND that allowed the data to be used for research	52 threads	8 (high)
							5 interviews	
Frost and Massagli (32)	Use of visual displays of health communication and conversations by people with ALS	Qualitative (content analysis)	PatientsLikeMe platform	Unknown	People living with ALS	ALS community on PatientsLikeMe	95 users with ALS	9 (high)
							123 postings	
Frost and Massagli (31)	Use of PatientsLikeMe by people with ALS in pulmonary health decision making	Qualitative (content analysis)	PatientsLikeMe platform	Unknown	People living with ALS	Posts and comments on the PatientsLikeMe ALS community including the words “trach” and ‘bipap’	• 395 members reporting non-invasive ventilation, 61 reporting tracheotomy and ventilation • Bipap: 583 forum posts, 26 comments, 907 private messages • Trach: 829 forum posts, 46 comments, 815 private messages	5 (medium)
Kazmer et al. (37)	Knowledge-building processes in online ALS community	Qualitative (content analysis)	PatientsLikeMe platform	Unknown	People affected by ALS	Random selection of posts in ALS community of PatientsLikeMe	241 individuals	8 (high)
							1,000 messages	
Hemsley and Palmer (34)	Feasibility and utility of Twitter data from adults with ALS and identify patterns in Twitter use	Qualitative (single case study and content analysis)	Twitter	Australia (based on ethical approval)	People living with ALS	Inclusion Tweets including #ALS and #MND	1 Twitter user with ALS for the case study	6 (medium)
							4,625 tweets for content analysis	
						Exclusion Tweets that were: duplicates; fundraising; tagged #ALSIceBucketChallenge or #StrikeOutALS		
Caron and Light (22)	Use and advantages of social media and barriers and facilitators to independent use for people with ALS,	Qualitative (asynchronous online focus group)	Social media/Wikispace	USA	People living with ALS	• ALS diagnosis • use of AAC for speech • independent use of at least one social media platform	11 people with ALS	9 (high)
							2 drop-outs → final sample size: 9	
							Age: 35–76	
							Male: *n* = 5	
							Female: *n* = 4	

### 3.2 Summary of interventions

All studies focused on text-based, asynchronous (not in real time) communication and covered different platforms. Discussion forums were covered most frequently (21, 33, 35, 36), followed by the PatientsLikeMe platform (31, 32, 37). Hemsley and Palmer (34) analysed Twitter, and Caron and Light (22) used a Wikispace for their online focus group. Most studies analysed a moderated platform, meaning that one or multiple people monitor the posts or facilitate the discussion (22, 31–33, 37), whereas others were unmoderated (34) or it was not specified (21, 35, 36) (Table 2).

**Table 2:** Online peer support characteristics.

Study + condition	Platform	Communication	Moderation	Reported outcomes	Successful elements	Implications
Stewart Loane and D'Alessandro (21)	Discussion forum	Text-based (asynchronous)	Unknown	• Majority of users was a patient, female, and reached out to the forum short after their diagnosis Social support: • Informational: most frequent • Network: second most frequent • Other: emotional, esteem, and instrumental • People initially join community to seek information, and are offered network and emotional support in addition	• Being part of an online peer community allows members to provide support to others as well. • This can be empowering, particularly for people who are highly dependent on others due to their condition	• Observation over a longer period • Specific attention to new vs. long-term members • Combination studies including observations, surveys, and participant interviews
Stewart Loane et al. (35)	Discussion forum	Text-based (asynchronous)	Unknown	Information support most frequent, followed by emotional support • Initial posts are often to request information, responses provide answers, and network and emotional support • When sharing info, the posters receive positive feedback • Spiritual support (expression of gratitude and feelings of connectedness) • Ethics/morality: participants refusing to provide a diagnosis or medical advice, but merely sharing personal experiences • Sharing poems and photos, humour, banter • Sense of community	• Patiens with ALS are highly dependent on others, but in an online community they can provide support to others, which can increase feelings of empowerment • ALS symptoms can limit a person's ability to fully participate in society in-person. Online communities overcome these barriers	• Using different methods to directly explore members’ experiences • Further explore what features of an online community promote a sense of community among members • Explore variety of online communities to identify whether specific features lead to greater value for members
Versteeg and te Molder (36)	Discussion forum	Text-based (asynchronous)	Unknown	• Members shared experiences and empathy • Members motivated each other to stay positive, be hopeful, and trust the medical/research community • Staying informed was seen as a moral duty	N/A	Exploring and understanding the patients’ needs can help to improve the relationship between patients and healthcare professionals
Hargreaves et al. (33)	Discussion forum	Text-based (asynchronous)	Charity staff	Themes: • Empathy through shared experiences • Reciprocity • Building friendships • Expression of feelings: language of empathy and cue for others to provide support • Empathy through connections (sense of belonging) • Space to share experiences and emotions • Feeling understood and less alone • Connection through similarity (symptoms, personal lives)	• Introductory posts to share story, starting point for conversation • Anonymous nature helped to be more open • Most found forum through own research (only 1 person was referred by a healthcare professional) • Members could create new spaces within forum	Explore: • barriers to expressing empathy online • impact of conflicts users and levels of sharing and empathy • relation of empathy on other aspects, e.g. self-disclosure or trust • role of privacy and trust in forum development Practice: • Raise awareness among healthcare professionals about online health communities • Make it easy for users to find specific info on forum • Allow different levels of communication (forum, one-to-one messages) • Interaction with other platforms (e.g. Facebook)
Frost and Massagli (32)	PatientsLikeMe	Text-based (asynchronous)	Yes	Comment categories: • Questions to others with relevant experience • Advice and recommendations • Relationship building • Comments lead to further conversations on discussion forum or private messages.	Technological features: • Graphical display of Gantt charts and images indicates length of illness, symptoms and treatments • Allow members to find others with relevant experience easily and to retrieve and provide tailored advice • Option to indicate geographical location: allowed members to connect and share info about local support	Explore the personal experiences of users through interviews and surveys
Frost and Massagli (31)	PatientsLikeMe	Text-based (asynchronous)	Yes	Members shared: • Advice on palliative care and assistive technologies for respiratory support • How they came to their palliative care decision • Views on end-of-life care and ALS progression	Technological features of forum: see Frost & Massagli (32) above.	Study the prevalence of each type of interaction and how it affects health outcomes
Kazmer et al. (37)	PatientsLikeMe	Text-based (asynchronous)	Yes	Distribution of knowledge: • in single thread: multiple users answer one question from another member • across threads: users referring to other relevant threads • across participants Creating knowledge: • Co-creating undiscovered public knowledge based on lived experiences • Co-creation of authoritative knowledge: combining medical literature with lived experience • Preference for lived experience • Other sources if no one has an answer	• Online, knowledge is distributed across geographical areas and time • When new members join, previously shared knowledge gets refreshed and they bring new knowledge. They can also identify new knowledge gaps • Technological feature: option to search for and link previous posts	• Role of technological design in distributing knowledge • Tools to streamline knowledge to support patients better • Use findings to design effective online platforms and encourage experts to join
Hemsley and Palmer (34)	Twitter	Text-based (asynchronous)	No	Study 1: • Most tweets were directed to individual Twitter users, including @ • Only 26% of the tweets included # and were directed to wider Twitter community • Content: ALS info, aspects of daily life, gratitude and emotions, social engagement and support. Study 2: • Main purpose of #s was to share Internet content (85%) • Conversational tweets (8%) included support, sympathy, concern, and encouragement • Status broadcast tweets, including hashtags such as #ALSsucks, #NeverGiveUp, related to raising awareness, creating one voice	• Majority of tweets would not come up when selecting data based on # only • Twitter is useful for people with ALS/MND and communication dissabilities	• Greater use of Twitter in future research for people with ALS and other conditions with communication disabilities • Explore lived experience of Twitter users with ALS or other conditions • Use study methods with larger groups
Caron and Light (22)	Wikispace	Text-based (asynchronous)	Researchers	Social connections: • Maintaining existing relationships • Reconnecting with people • Developing new connections Support network: • Reciprocal support • Raising awareness about ALS • Retrieving and sharing research-related info Communication opportunities: • Wider network to communicate with, reduced social isolation and loneliness Barriers: • Physical symptoms • Technological difficulties with AAC device	Social media • allows for communication beyond speech-based interaction • increases communication opportunities and access to support networks, and expanded social networks • overcomes challenges related to synchronous communication, for example through phone or video calls. The asynchronous nature allows people to communicate at their own pace	Recommendations from participants: • technology developers: allow flexible use AAC technology (indoors vs. outdoors, different websites) • policy makers: better support access to AAC technology • people with ALS: join social media and ALS groups Recommendations from authors: • Provide access to information about range of social media options • Provide access to appropriate supportive technologies • Provide people with knowledge and skills to use social media

### 3.3 Quality assessment

Six studies were of high quality (21, 22, 32, 33, 35, 37), and three were of medium quality (31, 34, 36). A table showing the results of the quality assessment for each individual study can be found in the Supplementary Material.

### 3.4 Key findings

An overview of the key findings is presented in Table 2. The main successful element identified in this review was social support, including informational, network, and emotional support.

#### 3.4.1 Benefits and challenges

Online peer support can be a convenient way of staying connected with others. Due to the nature of symptoms, people with ALS can experience difficulties in getting out of the house and meeting people. This can make the Internet a suitable alternative, as it offers different modes of communication and thus can support different needs and preferences. Being part of an online network can also create opportunities to get involved in advocacy and to raise awareness about ALS (22).

*One of the first abilities I began to lose was speech. Social events became more uncomfortable the worse my speech became. Even with the help of speech assistance [AAC support with speech output], group interaction is difficult. Facebook is a better communication tool for me. On Facebook we all are on the same level of communication ability* (22).

Only one study reported on challenges and potential barriers of online peer support (22). Physical symptoms of ALS can cause difficulties using a computer and typing. A potential solution could be eye-gaze technology. Furthermore, people may feel that online, text-based communication lacks emotion and body-language, and cannot replace real-life communication (22).

#### 3.4.2 Informational support

The Internet can offer a large amount of information on treatments, medication, and research opportunities (22). Frost and Massagli (32), Frost and Massagli (31), and Kazmer, Lustria (37) analysed the PatientsLikeMe platform, where users share symptoms, medications, and assistive tools they use through symbols on their profile. This can help people in identifying others in a similar situation or with relevant experience. When sharing advice and recommendations, people often shared personal experiences based on what others added to their profiles or asked targeted questions (32):

*I notice you have had a tube for about 8 months. I'm having difficulty eating so the neurologist suggested I look into getting one. It would help me if you would send me a message about your experience, pro and con* (32).

People used PatientsLikeMe to get advice on assistive technologies and discuss advance care planning and palliative care. They shared their experiences in deciding which type of assistive technology to use and practical hints and tips (31). For example, one person shared how they remain mobile while using a bipap machine:

*We put it on a small shelf behind the wheelchair and set the bipap on top of the battery […]. You plug your bipap in an inverter and plug the inverter into the battery. Very portable* (31).

Kazmer, Lustria (32) noted different people answered questions that were posted and signposted to other relevant threads on the platform. Threads had subject headings, for example “Loss of appetite from taking scopolamine”, making it easier to identify relevant topics. The option to search for information and previous discussion topics was experienced as helpful (37). Another benefit of asynchronous (not in real time) platforms is that people can ask for support or information when needed (22).

#### 3.4.3 Network support

Hargreaves, Bath (33) found that forum users perceived a real sense of community and support. This helped people talk about things that they would not necessarily feel comfortable speaking about with family or friends (22, 33).

*I have emotional lability […]. For those who understand, no explanation is necessary, for those who don't, no explanation is possible. Social media allows those emotional outbursts with no external discomfort. We can share in a place of understanding, in our own time and own pace without expectation or interruption* (22).

Being part of a network and supporting others can increase feelings of empowerment (21, 22). Through online peer support people can create value by sharing their experiences and advice (17). This is important, as people with ALS become increasingly reliant on others.

*I am so glad to find this site because I see there are many of us with slower progression than stereotypical. The support groups locally really focus on immediate need patients […]. It has been so great to see how long timers cope with losing our function slowly* (27).

#### 3.4.4 Emotional support

Through online platforms people shared empathy and compassion (21). People with ALS and their families try to have a positive outlook on things and shared this attitude by expressing empathy and support to others going through something difficult (36).

Hargreaves, Bath (33) discussed how it was for forum members when others dropped out because their ALS had progressed or they passed away. Sharing the grief over losing members of the forum, and losing the person someone once was created an emotional bond.

*There is a tremendous empathetic bond between the forumites. We share a life sentence. It cannot be more powerful than that. The feeling between us all on the forum has been strengthened through all these deaths. It is tangible* (33).

## 4 Discussion

### 4.1 Principal findings

This section presents the summary and interpretation of findings (narrative synthesis element 3: exploring relationships within and between studies). This review suggests that online peer support can be a valuable form of post-diagnostic support and has the potential to improve every domain of the Social Health Framework (9).

#### 4.1.1 Benefits and successful elements

This review shows that people with ALS use online peer support networks to exchange experiences and information. Learning from others can help people develop and improve coping skills and adapt to daily life with ALS (38). Online health communities, such as discussion forums and Facebook groups allow for a much larger membership than in-person groups, providing the opportunity to learn from a wide range of experiences. Wicks, Mack Thorley (38) found that PatientsLikeMe members reported improved feelings of control over their condition, and generally a better quality of life. This relates to two dimension of the Social Health Framework: (1) ability to fulfil potential and obligations, and (2) manage life with some level of independence (9).

Websites such as PatientsLikeMe allow people to share their experiences on their profile, making it easier to identify others in a similar situation or with relevant experience (31, 32, 37). Hargreaves, Bath (33) emphasize the importance of similarity. People indicated that having similarities stimulated conversation and that they felt more connected to those who share similarities with themselves (33). This supports earlier work by Lieberman, Wizlenberg (39) on online peer support for people with Parkinson's Disease. People with a similar age or time since diagnosis felt more connected to the people in their group and were less likely to drop out (39). Additionally, on asynchronous platforms information or discussion topics can be saved, allowing people to revisit what they find relevant (40, 41).

Furthermore, this review suggests that despite not being physically close, people with ALS can build meaningful connections and exchange support in an online setting. Online peer support can be a convenient way to connect with others from the comfort of one's own home, as ALS symptoms can make it more difficult to travel. This supports previous work by Leavitt, Riley (42), who found that people with Multiple Sclerosis felt safe and more comfortable joining online peer support compared to in-person groups. For people with ALS, difficulties with speech and experiencing emotional lability can make in-person events more challenging (22). Online peer support offers different forms of communication, tailoring towards different needs, abilities, and preferences. For example, asynchronous platforms allow people to communicate at their own pace and in their own time, without the need for verbal communication or the use of voice-assisted technologies. This relates to the last dimension of the Social Health Framework: being able to participate in social activities and work (9).

#### 4.1.2 Challenges

Physical symptoms of ALS can make it more challenging to use technological devices. Eye-gazing technology or AAC support could help, however, verbal and group interaction can remain challenging as it takes time to type on an AAC device, slowing down the communication (22). Asynchronous text-based platforms could offer a solution. However, previous research into online peer support on discussion forums shows that it can be difficult to judge the trustworthiness of online information (43, 44). A recent systematic review by Suarez-Lledo and Alvarez-Galvez (45) shows that on social media there is especially a risk for misinformation around health-related topics, including treatment, medication and interventions. In the context of misinformation, Turner (46) warns that through online peer support platforms, people can even be exposed harmful or misleading information. This raises the question of whether professionals should play a role in online patient communities by verifying and providing information, as has been considered for people with Multiple Sclerosis (20). However, including professionals might affect how freely speak to their peers, especially regarding their experiences with healthcare professionals. Additionally, facilitators and moderators have an important role in reminding people to always consult with their physician regarding medication, treatments, or symptoms (20).

### 4.2 Limitations

This section assesses the robustness of the synthesis (narrative synthesis element 4). Most studies included in this review used a qualitative content analysis methodology, and as a result this review only represents the views and experiences of people with ALS who are active on the online platform, meaning that they either create or respond to posts. However, research into online peer support for people with Multiple Sclerosis shows that non-active members can still benefit. The findings show that people can still feel a sense of community and benefit from practical hints and tips that others in the online group shared (47). Additionally, this review may over represent positive aspects of online peer support, since people who are active on an online platform tend to be the ones who enjoy it, only one study reported on the negatives and potential challenges of online peer support for people with ALS (22), and we could not include views and experiences of those who are unable to use or stopped using online peer support. Finally, with the qualitative content analysis methodology the findings can still remain dependent on the researchers’ interpretation (19).

### 4.3 Recommendations for future research

The systematic database search did not identify studies on verbal communication. During the COVID-19 pandemic videoconferencing platforms became increasingly popular. Nevertheless, after rerunning the database search no studies on using videoconferencing platforms for peer support were identified. However, as this review only represents the academic literature, some forums (e.g., Everything ALS and ALS ONE), Facebook groups, podcasts (e.g., Endpoint) and movements such as IamALS on Twitter are not represented. Furthermore, the use of PatientsLikeMe has declined. Future work could focus on the grey literature and real-world initiatives, to develop an up-to-date scoping review of online peer support communities for people with ALS. National ALS organisations could also provide such overviews on their websites, making it easier for patients to access this information and find a community that might suit them.

Despite the potential challenges with verbal communication, future research could explore whether videoconferencing platforms could be useful for peer support for people with ALS. Furthermore, previous research shows that text-based platforms have a large group non-active members, who still follow what is being shared (47). Future research could explore the experiences of this group, for example through surveys or interviews. Such research could also be used to gain knowledge on barriers for people with ALS to engage with technology or online peer support remains limited. Due to the progressive nature of ALS and the increasing challenges and barriers that people face, it is important to gain more insights in such barriers and how to overcome them, so that people can use online (peer support) resources for longer. With more aspects of health and social care being digitalised, a process that accelerated during the COVID-19 pandemic (48), it is important that people with ALS can engage with technology and use online communication systems. However, due to the progressive nature of symptoms, people with ALS may need support in using technological devices and engaging in online communication (49). Future research is needed to explore what kind of support people with ALS need in this, how it can best be embedded in health and social care systems. For example, future research could explore how the healthcare and voluntary sectors could support people with ALS in using technology, and how national governments and local healthcare commissioners can provide resources and support in this. While ALS is a rare condition, there are other conditions where people can experience symptoms that could impact their ability to use technology and engage in online communication, for example dementia (50), Parkinson's disease (19), and Multiple Sclerosis (20), which could benefit from such research as well.

## 5 Conclusions

Peer support can provide a supportive environment where people can connect and share experiences with others in a similar situation. This can help to develop adaptive coping skills. Online platforms can accommodate towards various needs, abilities, and preferences, as it offers different modes of communication. Particularly text-based, asynchronous (not in real time) platforms allow for people to engage at their own pace and in their own time, from the comfort of their own home. Such platforms can be especially useful for those who experience difficulties with verbal communication. However, ALS symptoms may make it more difficult for people to use technological devices and engage in online peer support. More research is needed to identify what kind of barriers people with ALS experience, and how these could be overcome.

## Data Availability

The original contributions presented in the study are included in the article/Supplementary Material, further inquiries can be directed to the corresponding author.
