# Supplementary material for: Online peer support for people with Amyotrophic Lateral Sclerosis (ALS): a narrative synthesis systematic review
Source: Front Digit Health. 2024 Jan 31;6:1138530. doi: 10.3389/fdgth.2024.1138530 (PMC10864493; doi:10.3389/fdgth.2024.1138530)
Supplement: Supplementary file 1 [file Table1.docx]

Supplementary Material

Online peer support for people with Amyotrophic Lateral Sclerosis (ALS): A narrative synthesis systematic review

Esther Vera Gerritzen^*^, Abigail Rebecca Lee, Orii McDermott, Neil Coulson, Martin Orrell

*** Correspondence:** [Esther.Gerritzen@nottingham.ac.uk](mailto:Esther.Gerritzen@nottingham.ac.uk)

Search terms

| **Search term 1** | **Search term 2** |
| --- | --- |
| Amyotrophic lateral sclerosis | online |
|  | digital |
|  | web-based |
|  | app-based |
|  | internet |
|  | social media |
|  | Peer |
|  | Peer support |
|  | Support group |
|  | Social support |
|  | Online support group |
|  | Online support commun* |
|  | Discussion forum* |
|  | Bulletin board |
|  | Chat room* |
|  | Computer-mediated support |
|  | Internet support group* |
|  | Internet support commun* |
|  | Online self-help |
|  | Web-based support group* |
|  | Web-based support commun* |

CASP checklist for Qualitative Studies

|  | Stewart Loane and D'Alessandro (2013) | Stewart Loane, Webster & D’Alessandro (2015) | Versteeg and te Molder (2019) | Hargreaves et al. (2018) | Frost & Massagli (2008) | Frost & Massagli (2009) | Kazmer et al. (2014) | Hemsley and Palmer (2016) | Caron and Light (2015) |
| --- | --- | --- | --- | --- | --- | --- | --- | --- | --- |
| 1 Clear statement of aims? | + | + | + | + | + | + | + | + | + |
| 2 Qualitative methodology appropriate? | + | + | + | + | + | + | + | + | + |
| 3 Research design appropriate? | + | + | + | + | + | + | + | + | + |
| 4 Recruitment strategy appropriate? * | + | + | - | + | + | - | + | + | + |
| 5 Data collected in a way that addressed the research issue? | + | + | + | + | + | + | + | + | + |
| 6 Has the relationship between researcher and participant** been adequately considered? | - | + | - | - | + | - | - | - | - |
| 7 Ethical issues been taken into consideration? | + | + | - | + | - | - | - | - | + |
| 8 Data analysis sufficiently rigorous? | + | + | + | + | + | + | + | + | + |
| 9 Clear statement of findings? | + | + | - | - | + | - | + | - | + |
| 10 How valuable is this research? | + | + | - | + | + | - | + | - | + |
| Total score | 9 | 10 | 5 | 8 | 9 | 5 | 8 | 6 | 9 |

+ = criterion met; – = criterion not met. *In case of qualitative content analysis this item focussed on selection of platforms and messages posted. **In case of qualitative content analysis ‘participants’ relates to the data analysis
